# Paraquat Exposure Up-regulates Cyclooxygenase-2 in the Lungs, Liver and Kidneys in Rats 

**DOI:** 10.22037/ijpr.2013.1358

**Published:** 2013

**Authors:** Hassan Malekinejad, Aysa Rezabakhsh, Fatemeh Rahmani, Mazdak Razi

**Affiliations:** a *Department of Pharmacology and Toxicology, Faculty of Veterinary Medicine, P. O. Box: 1177, Urmia University, Urmia, Iran. *; b *Department of Molecular Genetic, Faculty of Basic Sciences, Urmia University, Urmia, Iran. *; c *Department of Histology and Embryology, Faculty of Veterinary Medicine, Urmia University, Urmia, Iran. *

**Keywords:** Alveolar Edema, Cyclooxigenase-2, Liver, Multifocal Interstitial Nephritis, Paraquat

## Abstract

Paraquat is a commonly used herbicide in many countries which can lead to systematic poisoning on exposure, In this study, paraquat (PQ)-induced changes in the expression of Cyclooxygenase-2 (COX-2) along with biochemical and histopathological changes in the lungs, liver and kidneys were studied. Twenty four male Wistar rats (180-200 g) were exposed either against saline as control group or various doses of PQ (3.5, 7 and 10 mg/kg, SC) as test groups for 7 consecutive days. The animals in test groups demonstrated a significant increase of malondialdehyde and NO contents, while a remarkable decrease of total thiol molecules was recorded. Histopathological studies revealed a severe alveolar edema and hemorrhages in the lungs, congestion and glycogen degeneration in the liver and multifocal interstitial nephritis along with proximal tubular degeneration in the kidneys. PQ up-regulated the COX-2 expression at mRNA level significantly in the examined organs. This data suggest that the PQ-induced oxidative disturbances and pathological damages can be attributed to the upregulation of COX-2 in examined organs.

## Introduction

Paraquat (PQ; 1,1_-dimethyl-4,4_ bipyridium dichloride) is a cationic bipyridylium herbicide, which is frequently used as an effective, non-selective and non-systemic herbicide and exerts toxicity against eukaryotic organisms (1). Despite restricted usage in many countries, the occupational, intentional and accidental exposure of humans and the occupational and accidental exposure of animals still occurs (2). Its moderate and slight toxicity may occur via the oral and dermal routs of exposure in self poisoning and farmers, respectively (3). 

The well known mechanism of toxicity of PQ is the redox cycling as in this cycle PQ in dication form accepts an electron to form the monocation (PQ^+^) and then rapidly reacts with O_2_ to produce O_2_ -. and regenerate PQ^2+^ (4). It has been reported that the free radical producing mitochondrial NADH–quinone oxidoreductase is an oxidation–reduction system which mediates the PQ toxicity, suggesting that PQ-induced damages in mitochondria result in cell death (5). 

The main target tissue for PQ toxicity hitherto in mammalians has been reported to be the lungs due to its accumulation against a concentration gradient, via the polyamine uptake system that is abundantly expressed in the membrane of alveolar cells type I, II, and Clara cells (6). In this regard there are histopathological findings such as disruption, hemorrhages, edema and hypoxemia in alveolar epithelial and bronchiolar Clara cells, and proliferation of fibroblasts, excessive collagen deposition and infiltration of inflammatory cells into the interstitial and alveolar spaces (7). 

There are however, emerging data indicating that PQ exerts toxic effects in other organs such as liver and kidneys. We have shown that PQ up-regulates the predominant cytochrome P450 gene in the liver of rat, which may consequently induce detoxification processes (8). Moreover, another report showed that PQ in a dose-dependent fashion resulted in a hepatic antioxidant defense attenuation (9). Though, during the last decades numerous studies have been focused on PQ toxicity in various organs and all constantly confirmed that disturbing the antioxidant defense is the main mechanism of toxicity, however there is little known about the detail pathway(s) of PQ-induced inflammatory reactions in the target organs. Hence in this study in addition to the lungs as known target tissues, the PQ-induced oxidative injuries in the liver and kidneys were also investigated. To clarify the molecular mechanism of action of PQ in mentioned organs, the biochemical and histopathological changes were examined. Moreover, in order to show any inflammatory impact of PQ on selected organs and also to uncover the pathologically- and histochemically-induced injuries, relationship with molecular events, the mRNA level of inducible cyclooxygenase (COX-2) as a reliable biomarker of inflammation was determined. 

## Experimental

*Chemicals*

1, 1ʼ-dimethyl-4, 4ʼ-bipyridilium dichloride (Paraquat, PQ), sulfanilamide and N-(1-naphthyl) ethylenediamine. 2HCL were purchased from Sigma-Aldrich (Germany). Trizol was obtained from Invitrogen, Life Technologies (The Netherlands). Thiobarbituric acid, phosphoric acid (85%), dimethyl sulfoxide (DMSO), ethanol and sodium nitrite were obtained from Merck (Germany). N-butanol was obtained from Carl Roth, GmbH Co. (Germany). 

*Experimental animals*

This study was performed on 24 male and healthy Wistar rats, 10-12 weeks old, weighing between 180-200 g, kept at the animal center of Urmia University. The animals were kept in ventilated room at 22 ± 2 °C with a 12 h light/dark cycle. The rats were provided with food and water *ad libitum*. All performed experiments on animals were in accordance with the guidelines of the ethical committee for research on laboratory animals of Urmia University. 

*Experimental design*

Following a week acclimation, the animals were assigned into four groups (n = 6) as control and test groups. Before the experimental procedures, all animals were weighted and these procedures were repeated at the end of study to evaluate any changes in the body weight gain (BWG). Animals in the control group received physiological saline in a volume (0.5 mL/rat) similar to the PQ solution in the test groups. The animals in test groups received PQ (dissolved in sterilized saline normal) at the three various doses, representing low (3.5 mg/kg), medium (7 mg/kg) and high doses (10 mg/kg) of the PQ subcutaneously, for 7 consecutive days (10). 

*Serum preparation and tissue collection*

On day 8, immediately after a light anesthesia with diethyl ether, blood samples were collected directly from the heart and left to clot at room temperature for one h, and then centrifuged at 3000 ×g for 10 min to obtain the serum. After blood collection, the animals were euthanized using CO_2_ gas in special device and the lung, liver, and kidney tissues were dissected immediately. The collected tissue samples were divided into two parts and the first part after washing with chilled normal saline, were snap frozen in liquid nitrogen and then were immediately stored at -70 °C for further biochemical and molecular analysis. The second part of the samples was preserved in 10% buffered formaldehyde for further histopathological and histochemical examinations.

*NO measurement*

The total NO content of the lungs, liver and kidney samples was measured according to the Griess reaction (11). In Griess reaction, nitric oxide rapidly converts into the more stable nitrite and then in an acidic environment nitrite is converted to HNO_2_. In reaction with sulphanilamide, HNO_2_ forms a diazonium salt, which reacts with *N*-(1-naphthyl) ethylenediamine. 2HCl to form an azo dye that can be detected by absorbance at a wavelength of 540 nm. The NO content of the examined organs was expressed as nMol per mg of protein in samples. 

*Measurement of total thiol molecules (TTM)*

Total sulfhydryl levels in the lung, liver and kidney tissues were measured as described previously (12). Briefly, 0.3-0.4 g of the tissue samples were homogenized in ice-cold KCl (150 mM), and the mixture centrifuged at 3000 × g for 10 min. Then 0.2 mL of the supernatant of the tissue homogenate was added to 0.6 ml Tris-EDTA buffer (Tris base 0.25 M, EDTA 20 mM, pH 8.2) and thereafter 40 μL l 5.5’-Dithiobis-2-nitrobenzoic acid (10 mM in pure methanol) was added to the 10 mL glass test tube. The final volume of this mixture was made up to 4.0 mL by an extra addition of methanol. After 15 min incubation at room temperature, the samples were centrifuged (Roter-Uni II, BHG, Germany), at 3000 × g for 10 min and ultimately the absorbance of the supernatant was measured at 412 nm. The TTM capacity was expressed as nmol per mg of protein in samples. The protein content of the samples was measured according to the Lowry *et al*., method (13).

*Malondialdehyde (MDA) determination*

To determine the lipid peroxidation rate in the control and test groups, the MDA content of the lung, liver and kidney samples was measured using the thiobarbituric acid (TBA) reaction as described previously (14). Briefly, 0.2-0.3 g of the samples were homogenized in ice-cooled KCl (150 mM), and then the mixture was centrifuged at 3000×*g *for 10 min; 0.5 mL of the supernatant was mixed with 3 mL phosphoric acid (1% V/V) and then after vortex mixing, 2 mL of 6.7 g L^−1^ TBA was added to the samples. The samples were heated at 100 °C for 45 min, and then chilled on ice. After adding of 3 mL N-butanol, the samples were centrifuged at 3000×g for 10 min. The absorbance of supernatant was measured spectrophotometerically (Pharmacia, Novaspec II, Biochrom, England) at 532 nm and the amount calculated according to the simultaneously prepared calibration curve using MDA standards. The amount of MDA was expressed as nMol per mg protein. The protein content of the samples was assessed based of Lowery *et al*., method (13). 

*Histopathological and histochemical examinations*

Tissue samples from the lungs, liver and kidneys that previously had been stored in 10% buffered formaldehyde, were embedded in paraffin and 5-6 μm sections were cut using a rotary microtome and stained with Hematoxilin & Eosin for investigation under light microscope. In order to microscopically study lesions in examined organs, special histochemical staining of periodic acid Schiff (PAS) was conducted. To evaluate the level of damages following exposure to PQ, indices such as alveolar edema and hemorrhages, hepatic glycogen degeneration and congestion and renal multifocal interstitial nephritis and presence of protein cast in renal tubules were scored numerically. The evaluation criteria were as follows: zero for no detectable lesion, 1 for mild changes, 2 for moderate changes and 3 for severe changes. For each animal in the tests and control groups, at least three slides from distinct organs were prepared and scored.

*RNA isolation and RT-PCR*

To evaluate the effect of PQ on COX-2 mRNA level, tissue samples from the lung, liver and kidney were collected and snap frozen in liquid nitrogen and then stored at -70 °C until RNA isolation. Total RNA was isolated from pooled samples homogenates for the control and test groups (n = 6) using the standard TRIZOL method (15). To avoid genomic DNA contamination, extra care was taken when the colorless aqueous phase was collected after chloroform extraction. The RNA amount was determined spectrophotometrically (260 nm and A260/280=1.8-2.0), and the samples were stored at -70 °C. For RT-PCR, cDNA was synthesized in a 20 μL reaction mixture containing 1 μg RNA, oligo(dT) primer (1 μL), 5×reaction buffer (4 μL), RNAse inhibitor (1 μL), 10mM dNTP mix (2 μL) and M-MuLV Reverse Transcriptase (1 μL) according to the manufacturer’s protocol (Fermentas). The cycling protocol for 20 μL reaction mix was 5 min at 65 °C, followed by 60 min at 42 °C, and 5 min at 70 °C to terminate the reaction. We ran the PCR reaction with using specific RT-PCR primers –stated below- on RNA samples and no PCR bands were observed indicating that the RNA samples are free from genomic DNA.

*Second strand cDNA synthesis*

The RT-PCR reaction was carried out in a total volume of 25 μL containing PCR master mix (12.5 μL), FWD and REV specific primers (each 0.5 μL) and cDNA as a template (1 μL) and nuclease free water (10.5 μL). PCR conditions were run as follows: general denaturation at 95 °C for 3 min, 1 cycle, followed by 40 cycles of 95 °C for 20s; annealing temperature (62 °C for *β*-actin and 59 °C for COX-2) for 30 s; elongation: 72 °C for 1 min and 72 °C for 5 min. To show the lack of any genomic DNA contamination, PCR reaction was conducted on cDNA samples with two forward and reverse primers designed from intron regions of COX-2 and no PCR bands were observed on the gel, while running PCR with these two primers on genomic DNA sample extracted from rat liver, showed a 207 bp PCR fragment as we expected. 

The products of RT-PCR were separated on 1.5 % agarose gels containing ethidium bromide and visualized using Gel Doc 2000 system (Bio-Rad). The density of RT-PCR bands were quantified by using the software of Molecular Analyst (Bio-Rad, the Netherlands) and normalized based on the density of corresponding *β*-actin bands. The RT-PCR reaction and subsequent electrophoresis were performed three times and the averages of numerical densitometric values along with standard deviation were calculated. The specific primers for Ratus COX-2 and *β*-actin were designed (16) and manufactured by CinnaGen (CinnaGen Co. Tehran, Iran). 

Primers pairs for RT-PCR were as follow:

COX-2 (the expected PCR product size = 252 bp): 

Forward primer 5ʼ-TGGTGCCGGGTCTGATGATG-3ʼ and 

Reverse primer 5’-GCAATGCGGTTCTGATACTG-3’, 

*β*-actin (The expected PCR product size = 399 bp):

Forward primer 5′-CTGACCGAGCGTGGCTACAG-3′ and 

Reverse primer 5′-GGTGCTAGGAGCCAGGGCAG-3′). 

*Statistical analysis*

The mean and standard deviations of measured parameters were calculated. The results of three independent experiments for each assessment were analyzed using Graph Pad Prism software (version 2.01; Graph Pad software Inc. San Diego, California). The comparisons between groups were made by the analysis of variance (ANOVA) followed by Bonferroni post hoc test. For comparing the graded degree of pathological findings between groups, the Kruskal-Wallis test was used. A p-value < 0.05 was considered significant.

## Results

*PQ exposure resulted in body weight loss*

The clinical symptoms such as nasal and eyes bloody discharges along with difficult breathing were observed in PQ-exposed animals. Moreover, exposed animals demonstrated a dose-dependent body weight loss as 10.3 ± 1.5, 11.3 ± 3.2, and 16.0 ± 3.0 g body weight which was recorded in animals that received low, medium and high dose levels of PQ, respectively. While during the 7 days study period the control group showed 21.3 ± 5.5 g body weight gain. 

*PQ exposure enhanced the no and MDA contents and attenuated the TTM level*

**Figure 1:**
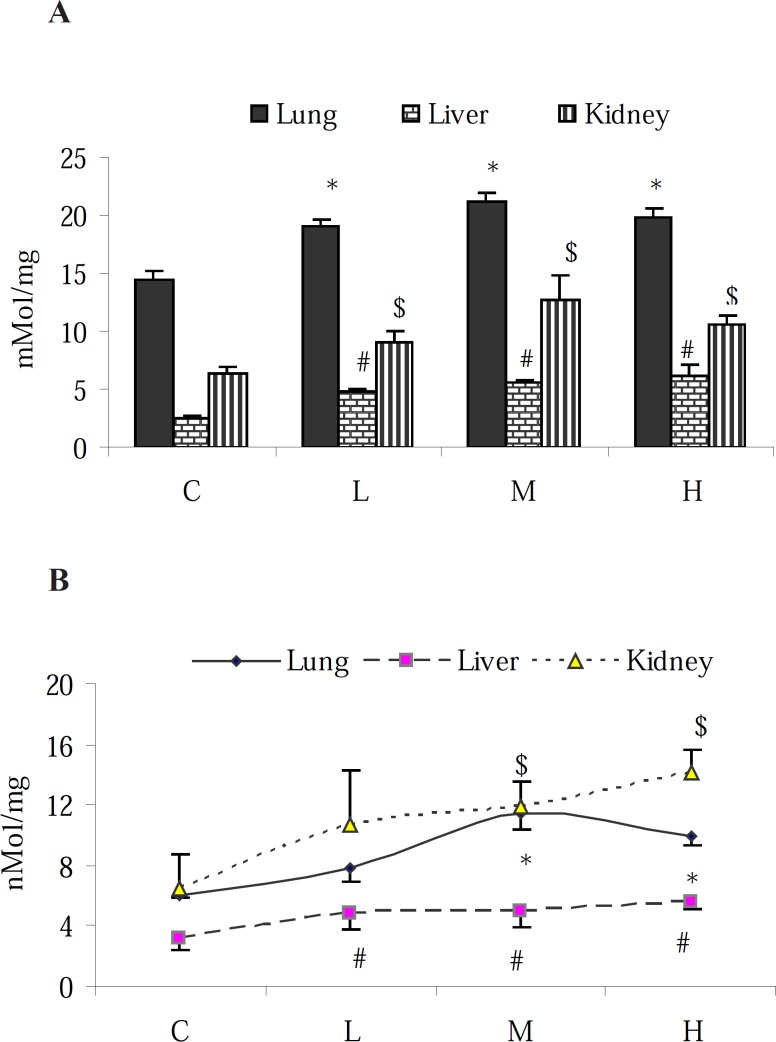
Effect of paraquat on: (A) NO content and (B) MDA level in the lungs, liver and kidneys; bars represent mean ± SD; n = 6 in each group. Stars, #, and $ indicate a significant (p < 0.05) difference between the control and PQ-exposed groups in the lungs, liver and kidneys, respectively. C= control (received vehicle); L = low dose (3.5 mg/kg), M = medium dose (7 mg/kg) and H = high dose (10 mg/kg).

Results of NO-content determination in proposed tissues revealed that in the control animals, the highest level of NO was found in the lungs followed by the kidney and liver. PQ elevated the NO-content of all examined organs (Figure 1-A). Although the elevation of NO level in the liver was in a dose-dependent manner, in the lung and kidney, however the highest level of NO was found in the animals that received the medium dose level (7 mg/kg) of PQ. To measure the pro-oxidant effect of PQ in exposed animals, MDA content of the tissue samples were assessed. The obtained results showed an increase of MDA content in the tested organs (p < 0.05) with the highest increase in the kidney (Figure 1-B). 

**Figure 2:**
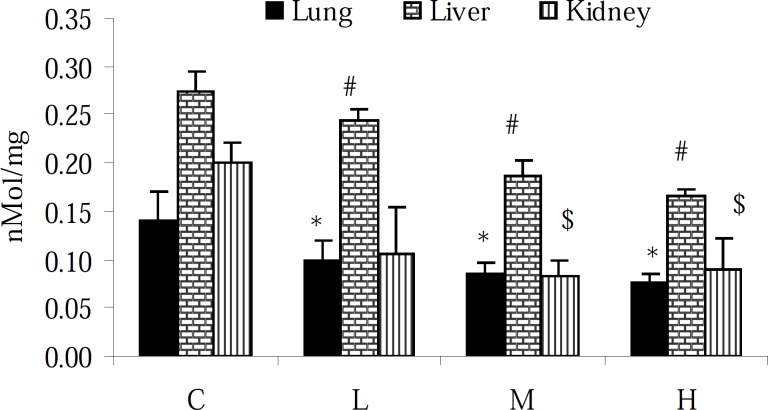
Effect of paraquat on TTM level in the lungs, liver and kidneys; bars represent mean ± SD; n = 6 in each group. Stars, #, and $ indicate a significant (p < 0.05) difference between the control and PQ-exposed groups in the lungs, liver and kidneys, respectively. C= control (received vehicle); L = low dose (3.5 mg/kg), M = medium dose (7 mg/kg) and H = high dose (10 mg/kg).

Assessment of the TTM level in the lung, liver and kidney of intact animals showed that the TTM content of the liver was approximately 1- and 0.5-fold higher than that in the lung and kidney tissues, respectively. Moreover, 7 days exposure to PQ resulted in the reduction of the TTM content (p < 0.05) in all tested tissues (Figure 2). 

*Pathological findings*

**Figure 3:**
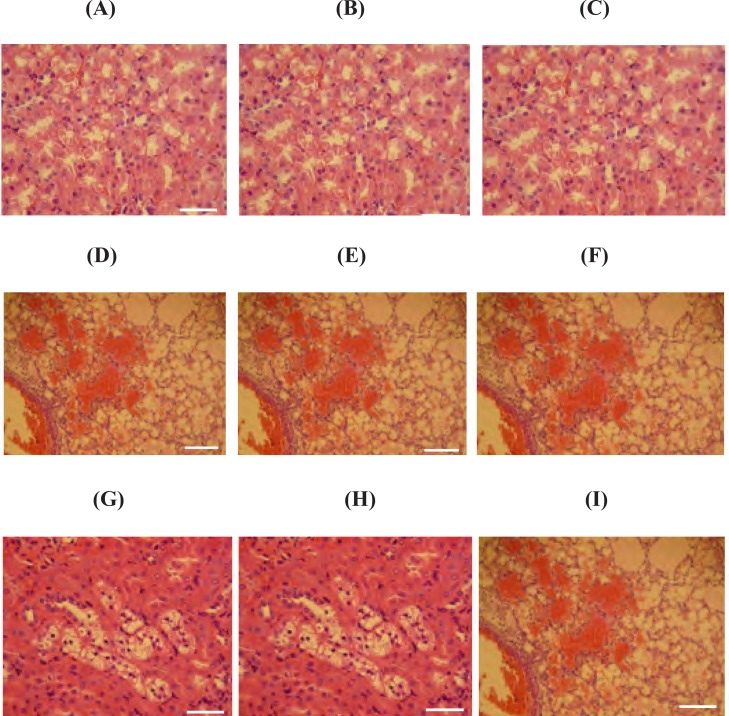
Photomicrograph of rat's lung, liver and kidney sections; (A, B and C; 400 x) representing the lung, liver and kidney from control group; no histological changes are observed, (D and G; 100 and 400 x respectively) lungs of PQ-exposed rats at medium and high doses respectively; a severe alveolar edema and hemorrhages is seen, (E and H; 100 and 400 x respectively) liver of PQ-exposed rats at medium and high doses respectively; representing a severe congestion along with swelling of hepatocytes and the fatty degeneration in periportal zone of classical lobules and (F and I; 100 and 400 x respectively) show the kidney from animals that exposed to medium and high doses of PQ respectively; multifocal interstitial nephritis, and the presence of protein casts in renal tubules are manifested. E&H staining and scale bar is 0.1 mm

The normal histological feature of the lungs, liver and kidney from the healthy rats are depicted in Figures 3-A, 3-B and 3-C. Histopathological findings in the lungs of PQ-exposed animals represented severe hemorrhages, marked congestion, alveolar edema, emphysema and remarkable infiltration of neutrophils and lymphocytes in interstitial tissue (Figure 3-D and 3-G). Moreover, histopathological findings in the liver of test groups showed a severe congestion along with swelling of hepatocytes and consequently reduction of sinusoidal spaces. We also observed the fatty degeneration in periportal zone of classical lobules (Figure 3-E and 3-H). Additionally, in the kidneys, pathological changes such as congestion, tubular swelling and necrosis, multifocal interstitial nephritis, and the presence of protein casts in renal tubules were seen (Figure 3-F and 3-I). The severity of histopathological damages is shown as numerical data in Table 1. 

**Table 1:** Pathological findings in the lungs, liver and kidneys after 7 days exposure to PQ; mean values ± SD are given

**Groups**	**AE&H**	**HGD&C**	**MIN&PC**
C	0.0 ^a^	0.0 ^a^	0.0 ^a^
L	1.8 ± 0.3 ^b^	1.2 ± 0.4 ^b^	1.8 ± 0.6 ^b^
M	4.2 ± 0.6 ^c^	3.2 ± 0.4 ^c^	4.6 ± 0.8 ^c^
H	6.2 ± 1.2 ^d^	7.8 ± 1.8 ^d^	7.8 ± 1.2 ^d^

abcd: values in same column with different superscripts differ significantly ( p < 0.05). indexes such as alveolar edema and hemorrhages (AE&H), hepatic glycogen degeneration and congestion (HGD&C) and renal multifocal interstitial nephritis and presence of protein cast in renal tubules (MIN&PC), C= control (received vehicle); L = low dose (3.5 mg/kg), M = medium dose (7 mg/kg) and H = high dose (10 mg/kg).

**Figure 4:**
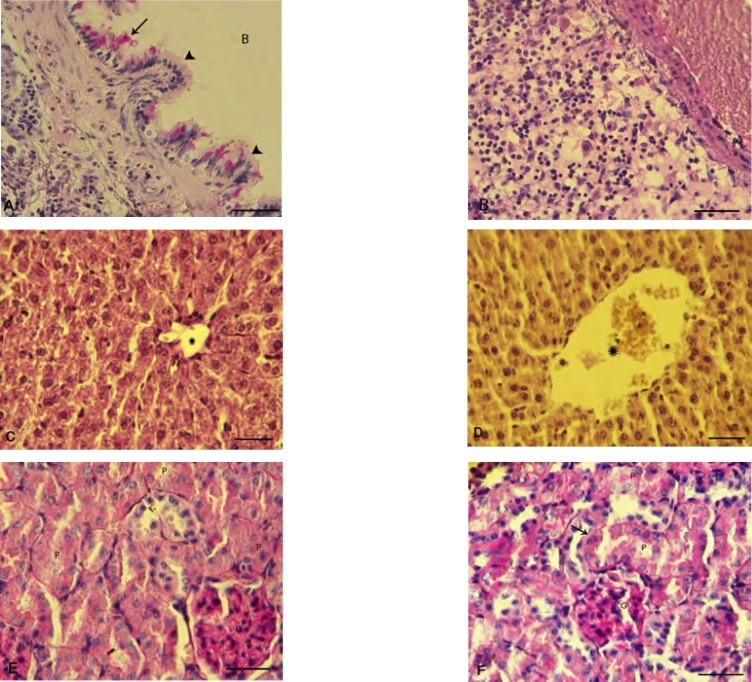
Photomicrograph of rat's lung, liver and kidneys: (A) normal bronchiole cross section with intact columnar epithelium (*head arrows*) and glycoprotein secretion (*arrow*), (B) inflammatory cells infiltration in the lung of PQ-exposed animals, (C) intact liver is showing the normal glycogen supplemention with positive PAS stained cytoplasm. Star is representing normal portal vein diameter. (D) Glycogen degeneration is demonstrated in hepatocytes of the PQ-exposed animals with negative reaction for PAS staining and star is presenting dilated portal vein. (E) Intact kidney shows the normal proximal tubules (*P*) and glomerulus (*G*) and (F) PQ-exposed animals represent an impaired kidney with diffused proximal tubules degeneration (*P*), increased thickness of basement membrane (arrow) and degenerated glumerulus (*G*). Periodic acid shift staining, (400X) and scale bar is 0.2 mm

To confirm the details of the PQ-induced damages in the examined organs, the histochemical staining of PAS was carried out and the observations revealed that in the lung tissue PQ-exposure (10 mg/kg) resulted in a clear and severe edema, infiltration of inflammatory cells in particular around the dilated vessels (Figure 4-A and 4-B). 

Histochemical staining on the liver and kidneys also were performed. In the control group, a positive PAS stained cytoplasm and normal portal vein were observed. However in PQ-exposed animals, the glycogen degeneration in hepatocytes with negative reaction for PAS was seen (Figure 4-C and D). The identified lesions in the kidney were indicative of the proximal tubules degeneration, an increase in basement membrane thickness and degenerated glomerulus in PQ-exposed animals (Figure 4-E and 4-F).

*Paraquat up- regulated the mRNA level of COX-2*

**Figure 5:**
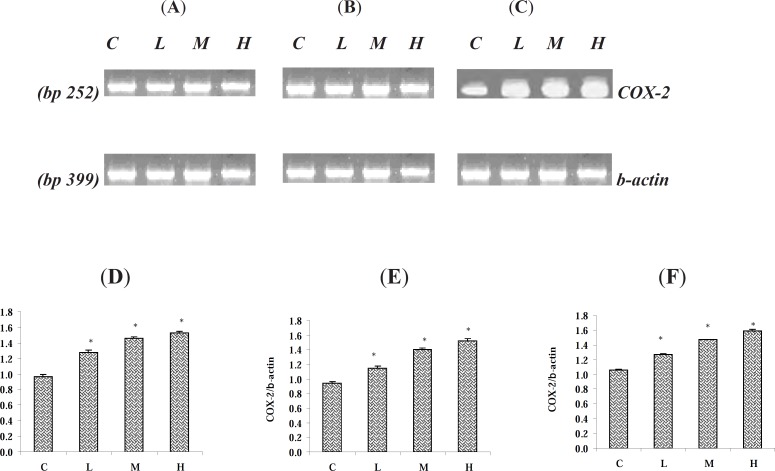
Effect of Paraquat on COX-2 mRNA level in: (A) the lungs, (B) the liver, and (C) the kidneys; the expression of *β*- actin as control gene from the corresponding animals are depicted in lower panels. The levels of COX-2 mRNA were evaluated by semi-quantitative RT-PCR. Lower panels represent the COX-2 mRNA levels in: (D) the lungs, (E) the liver and (F) the kidneys; that were measured by densitometry and normalized to *β*-actin mRNA expression level. Results were expressed as integrated density values (IDV) of COX-2 mRNA level. C= control (received vehicle); L = low dose (3.5 mg/kg), M = medium dose (7 mg/kg) and H = high dose (10 mg/kg).

Following 7 days exposure of the rats to various doses of PQ, the mRNA level of COX-2 and *β*-actin were measured using RT-PCR method. We found that in all three examined tissues albeit with minor differences, the COX-2 gene was expressed. The mRNA level of COX-2 in the tested tissues was up regulated (p < 0.05). Though the up regulation of COX-2 gene was dose-dependent as we found the highest level of up regulation of COX-2 at the highest given dose level of PQ in the examined organs (Figure 5-A, 5-B and 5-C). The expression of *β*-actin gene as control gene is depicted in figures 5-A, 5-B, and 5-C. The levels of mRNA were expressed as the ratio of integrated density values (IDV) of COX-2 mRNA level to *β*-actin mRNA levels (Figure 5-D, 5-E and 5-F). 

## Discussion

This study reports an upregulation of COX-2 in the lungs, liver and kidneys following exposure to PQ. Moreover, we demonstrated that in addition to oxidative disturbances, nitrosative stress also mediates the toxicity of PQ. 

The most pathological impact of PQ intoxication has been reported in the lungs. There are emerging data indicating that other organs including the liver and kidneys may also be affected from PQ-induced toxicity (17-19). Our results uncovered the PQ-induced biochemical changes including oxidative/nitrosative markers alterations and pathological injuries in the liver and kidneys. Therefore, it might be worth to consider the hepatic- and renal-related biochemical changes as biomarkers of PQ intoxication. We recently showed that PQ altered the serum level of alkaline phosphatase (8). Moreover, in current study other biomarkers such as hepatic and renal NO and TTM levels alterations due to PQ intoxication were induced. 

The major role of NO in PQ-mediated neurotoxicity and up regulation of iNOS in leukocytes of PQ-exposed rats has been demonstrated (19 -21). Previous studies on lung epithelial cells showed that following exposure to PQ, the NO formation was elevated (22). Our results indicate that NO increase in the liver and kidney might play a key role in PQ-mediated hepato- and renotoxicity. This finding is supported by enhanced level of MDA, suggesting that the NO elevation resulted in further lipid peroxidation. Our findings are in agreement with previous reports showing that antioxidant quercetin was able to reduce the PQ-induced MDA production and NO increase in the lungs (23). The PQ exposure resulted in a reduction of TTM in target organs as an indicator of intracellular antioxidant capacity (24).

We report here new histopathological findings in the lungs of PQ-exposed animals such as hemorrhages, alveolar edema, emphysema and infiltration of neutrophils and lymphocytes in interstitial tissue, which have not been described with previous reports (22-26). On the other hand, the pathological injuries such as severe congestion, hepatocyte swelling, glycogen degeneration in the liver and interstitial nephritis along with degeneration of proximal tubules, all indicates inflammatory reactions in either organs. Increase in inflammatory mediators such as NO and decrease in glutathione levels confirm the pathological findings and may play role in the initiation of observed pathological injuries. The presence of inflammatory cells such as neutrophils and lymphocytes may promote the inflammatory reactions as the role of mononuclear inflammatory cells in producing NO and other inflammatory cytokines has been reported (27). 

An interesting finding of this study is that PQ-exposure up-regulates the COX-2 mRNA level in the examined organs. Very recently PQ-induced toxicity in dopaminergic neurons has been reported. This *in-vitro *model demonstrated that in human neuroblastoma SH-SY5Y cells, upregulation of COX-2 due to PQ exposure is mediated via NF-κB activation (28). Although the activation of NF-κB in the examined tissues is not excluded, however it seems that the toxic effects of PQ such as the total thiol molecules reduction, lipid peroxidation and NO level elevation more likely related to the COX-2 up regulation. The inflammatory reactions which have been described in the studied organs, may also be associated to COX-2 upregulation. It has also been demonstrated that COX-2 is expressed in all three tested organs (29). Therefore, it would be worth if it is hypothesized that PQ, by disturbing the antioxidant defense results in a release of pro-inflammatory factors such as NO, which in turn leads to inflammation. Our findings support this hypothesis as a significant upregulation of COX-2 in the tested organs confirms an occurrence of inflammation that accompanied the biochemical and histopathological inflammatory changes. 

## Conclusion

These findings may help to understand the pathogenesis of PQ-induced damages and lead us to consider a new regimen of therapy in the management of PQ poisoning. Moreover, another reliable biomarker of COX-2 up-regulation could be applied in the precise diagnosis of PQ-poisoned cases. 
