# Expression of Concern for Paraquat Exposure Up-regulates Cyclooxygenase-2 in the Lungs, Liver, and Kidneys in Rats [Iran J Pharm Res. 2013; 12(4): e125760]

**DOI:** 10.5812/ijpr-151659

**Published:** 2024-10-28

**Authors:** Editor-in-Chief IJPR

**Affiliations:** 1 School of Pharmacy, Shahid Beheshti University of Medical Sciences, Tehran, Iran

The Ethics Committee of Brieflands is issuing this Expression of Concern regarding the article titled "Paraquat Exposure Up-regulates Cyclooxygenase-2 in the Lungs, Liver, and Kidneys in Rats," which was published in the Iranian Journal of Pharmaceutical Research (1).

We have been alerted to potential issues related to image manipulation and data fabrication in Figures 3 and 5 of the aforementioned article. These concerns were reported to us by a whistleblower via ticket #534602, who provided a detailed analysis and evidence supporting these claims.

In light of these serious concerns, we are currently conducting a thorough investigation to determine the validity of these allegations. During this period, readers are advised to interpret the findings of the article with caution.

We will provide an update with the results of our investigation and any necessary actions that may follow, including potential retraction of the article if the concerns are substantiated.

We take issues of scientific integrity very seriously and are committed to ensuring the accuracy and reliability of the research published in our journal.

Thank you for your understanding and patience as we address this matter.

Sincerely,

Head of the Ethics Committee 

Brieflands
